# Differential expression and functional analysis of circular RNAs and m6A modifications in children with Philadelphia chromosome-positive acute lymphoblastic leukemia

**DOI:** 10.1038/s41598-025-97345-0

**Published:** 2025-04-24

**Authors:** Lichun Xie, Ye Xu, Chuiqin Fan, Maochuan Liao, Guichi Zhou, Chen Fen, Lian Ma, Fei-Qiu Wen

**Affiliations:** 1 Department of Obstetrics and Gynecology, Department of Paediatrics, Guangdong Provincial Key Laboratory of Major Obstetric Diseases, Guangdong Provincial Clinical Research Center for Obstetrics and Gynecology, Guangdong-Hong Kong-Macao Greater Bay Area Higher Education Joint Laboratory of Maternal-Fetal Medicine; The Third Affiliated Hospital, Guangzhou Medical University, Guangzhou, 510140 Guangdong China; 2 Department of Haematology/Oncology, Shenzhen Children’s Hospital, No. 7019 Yitian Rd, Shenzhen, Guangdong China

**Keywords:** Circular RNA, Philadelphia chromosome-positive acute lymphoblastic leukaemia, N6-methyladenosine, Cancer, Haematological cancer

## Abstract

Philadelphia chromosome-positive (Ph^+^) acute lymphoblastic leukaemia (ALL) in childhood is associated with dismal outcomes, in large part due to challenges in diagnosis and monitoring therapeutic efficacy. Recent studies suggest that circular RNAs (circRNAs) are potential diagnostic and prognostic biomarkers for various tumours. to indicate the potential role of circRNAs in identifying or serving as novel targets for treatments. Here, we analysed CircRNA expression profiles in samples from three Ph^+^ ALL patients at diagnosis (CK1 group), on day 19 after treatment (T1 group) and in first complete remission (day 46 after treatment, T2 group), as well as one Ph^−^ ALL patient at diagnosis (CK2 group). A total of 922 differentially expressed circRNAs (DECs) potentially associated with RNA degradation, microRNAs in cancer, propanoate metabolism and ubiquitin-mediated proteolysis were found (626 upregulated and 296 downregulated) between the CK1 and CK2 groups. In addition, we identified 224 DECs (129 upregulated and 95 downregulated) between the CK1 and T1 groups and 225 DECs (136 upregulated and 89 downregulated) between the CK1 and T2 groups, including 136 for which their expression was upregulated and 89 for which their expression was downregulated. The levels of hsa_circ_0012152 and hsa_circ_0009024 were significantly increased in Ph^+^ ALL patients, the changes in the levels of these circRNAs were confirmed by qRT‒PCR, indicating their potential as diagnostic biomarkers. Most upregulated DECs underwent N6-methyladenosine (m6A) modification noting the specific roles that are now better understood based on the circRNAs and DECs identified, and ideally suggesting how the findings could impact the diagnosis and treatment of Ph^+^ ALL The findings of this study increase our understanding of the roles of m6A-modified circRNAs in the pathogenesis of Ph^+^ ALL.

## Introduction

Acute lymphoblastic leukaemia (ALL) is the most frequently occurring malignancy in children and also ranks first in terms of cancer-related death in children worldwide^1^. Approximately 3–5% of children with ALL possess a t (9;22) (q34;q11.2) translocation, also known as the Philadelphia chromosome (Ph), which results in the production of the BCR-ABL1 oncoprotein^2^. In children, Ph^+^ ALL is associated with dismal outcomes, including high rates of relapse and poor long-term survival. Before the advent of targeted drugs, haematopoietic stem cell transplantation (HSCT) was the best curative option for Ph^+^ ALL, but fewer than half of patients who underwent HCST survived. Although the combination of targeted BCR-ABL1 inhibition with chemotherapy has improved the prognosis of Ph^+^ ALL patients, patients with Ph^+^ ALL often develop resistance to treatment and are at high risk of relapse, and the outcomes of R/R ALL patients treated with single‐agent tyrosine kinase inhibitors (TKIs) are poor. Therefore, new diagnostic and prognostic biomarkers and therapeutic targets for Ph^+^ ALL are needed to aid the development of more effective disease monitoring and treatment programmes^3^.

ALL has been proven to be a heterogeneous disease. The initiation and progression of ALL is a complex pathological process. There are > 20 subtypes of B-cell ALL (B-ALL), which are associated with distinct gene expression profiles and are driven by three main types of initiating genetic alterations. Each subtype typically has cooccurring genetic alterations that perturb lymphoid development, cell cycle regulation, kinase signalling and chromatin regulation; however, the genes involved and the frequency of their involvement vary among subtypes^4^.

Research has increasingly suggested that various types of biological molecules, including noncoding RNAs, play important roles in this process. For example, studies have revealed that circular RNAs (circRNAs), each of which is comprised of a single RNA molecule with covalently linked ends, are more stable than linear RNAs. To date, many circRNAs have been identified in different cell lines and species. CircRNAs can drive cancer development by acting as miRNA and RNA-binding protein sponges or by encoding polypeptides or proteins to participate in the proliferation and invasion of tumour cells and in biological processes such as metastasis, apoptosis, and epithelial‒mesenchymal transition^5^, and the role of methylation of the N6 position of adenosine (m6A) in regulating circRNA function in these processes is also gradually becoming clear^6^. Specifically, several circRNAs have been implicated in haematopoietic malignancies, such as acute myeloid leukaemia (AML)^7^, mixed lineage leukaemia (MLL)^8^-rearranged leukaemia and T-cell ALL (T-ALL). However, to date, the circRNAs expressed in the bone marrow of children with Ph^+^ B-ALL have not been characterized and the lack of knowledge about the difference between circRNA profiles (including m6A modification) of Ph + and Ph- B-ALL or the changes in circRNA profiles associated with treatment response^9^.

Therefore, the aim of this study was to identify therapeutic targets specifically for childhood Ph + ALL, or elucidating the pathogenic mechanism of childhood Ph^+^ ALL whether there are differences in circRNAs expression profiles in the bone marrow between children with Ph^+^ B-ALL and Ph^−^ B-ALL at the time of diagnosis or between children with Ph^+^ B-ALL before and after treatment. We identified differences in circRNA expression profiles between children with Ph^+^ B-ALL and those with Ph^−^ B-ALL and among children with Ph^+^ B-ALL at different time points were studied. Our findings identify upregulated and m6A-modified circRNAs with potential roles in Ph^+^ B-ALL pathogenesis that may have clinical value as biomarkers or future targets. The identified differentially expressed m6A-modified circRNAs may be used to improve diagnosis, prognostic prediction, and treatment.

## Methods

### Patient and sample selection

A total of four patients newly diagnosed with Ph^+^ B-ALL or Ph^−^ B-ALL were included in this study. All patients were treated according to the protocol of the Chinese Children’s Cancer Group study ALL-2015 (CCCG-ALL-2015). Bone marrow samples were collected from 3 patients with Ph^+^ B-ALL at three time points: at diagnosis (CK1 group), on day 19 (T1 group), and at first complete remission (day 46, T2 group) after treatment. One patient with Ph^−^ B-ALL provided a bone marrow sample at diagnosis (CK2 group). This study was conducted in accordance with the principles of the Declaration of Helsinki. Ethical approval was obtained from the ethics committee of Shenzhen Children’s Hospital, and informed consent was obtained from the parents of each participant. The clinical data are provided in Table 1.

**Table 1:** Clinical information for the bone marrow samples.

Group	Risk level	Fusion gene(s)
CK1	Intermediate risk	BCR-ABL
CK1	Intermediate risk	BCR-ABL
CK1	Intermediate risk	BCR-ABL
CK2	Low risk	43 recessive fusion genes

### Cell lines

A human Ph^+^ B-ALL cell line (Sup-B15 cell line) and a Ph^−^ B-ALL cell line (Nalm-6 cell line) were purchased from the Chinese Academy of Science (Shanghai, China). All the cells were cultured in Dulbecco’s modified Eagle’s medium supplemented with 10% foetal bovine serum. All the cells were grown at 37 °C in 5% CO_2_.

### RNA extraction from bone marrow aspirates

Total RNA was extracted from each sample with a TRIzol reagent kit (Invitrogen, CA, USA), and the RNA concentration, purity and integrity were assessed with an Agilent 2100 Bioanalyzer according to the manufacturer’s protocol (Agilent Technologies, CA, USA). Then, the RNA was purified and reverse transcribed into cDNA with the SuperScript™ IV First-Strand Synthesis System (Invitrogen, CA, USA) according to the manufacturer’s instructions. CircRNA expression levels were measured via quantitative real-time quantitative PCR (qRT‒PCR).

### Library construction and sequencing

A strand-specific library was constructed with the VAHTS Total RNA-seq (H/M/R) Library Prep Kit (Vazyme, Nanjing, China) for Illumina following the manufacturer’s instructions. RNA sequencing was performed by Genedenovo Biotechnology Co., Ltd. (Guangzhou, China) using the Illumina NovaSeq 6000 sequencing platform, and differentially expressed circRNAs (DECs) in bone marrow aspirates were identified using the edgeR package on the basis of a fold change (FC) ≥ 2 and a P value < 0.05. The expression levels of these circRNAs in B-ALL cell lines were verified via qRT‒PCR. The sequences of the primers used for qRT‒PCR are listed in Table 2. The relative expression of target gene A was calculated via the 2-ΔΔCt method as follows: ΔCt = Ct(A)−Ct(B) and − ΔΔCt = ΔCt(control group)−ΔCt (experimental group). Summarization, normalization, and quality control of the data were performed.

**Table 2:** Primer sequences.

Primer name	Forward primer	Reverse primer
hsa_circ_0012152	AGCCCAGAGGATCGGAATGA	CGTTACGCAAGAAGCAGTCT
hsa_circ_0009024	CTGAGCAAGAAGTAGCCCCTC	CCACAAAGTCCAGCTTCTTCC
hsa_circ_0002754	ACCAACGTGGATGGGAAAGA	TCCCCAAGAAACTAGTCAGCAC
hsa_circ_002156	TTGGACCTCAGTGTTGTGGA	CCAACTGCAAATTGTTCTGC
hsa_circ_0017627	CCCAGGAATCCAGGAGAACT	GGAAATTTGGCAGCATCAAT
hsa_circ_000525	TAAACTTGAAAGGCACCGCAG	CTCTCCATGCCTGATGAGCAA
hsa-GAPDH	AGAAGGCTGGGGCTCATTTG	AGGGGCCATCCACAGTCTTC

### Bioinformatic analysis

Bioinformatic analysis was conducted to evaluate the diagnostic and prognostic accuracy of the identified significantly DECs as potential candidate biomarker(s). Enrichment analysis of the target genes of the DECs was performed via DAVID to predict the function of the circRNAs.

### Prediction of m6A sites with SRAMP

The potential m6A sites of the top 20 DECs were predicted via SRAMP (http://www.cuilab.cn/sramp/)^10^. Mature mRNA mode with a generic predictive model was utilized, the RNA secondary structure was analysed, and RNA was selected as the query sequence. The results are shown as the prediction score distribution along the query sequence. The prediction scores included very high confidence, high confidence, moderate confidence and low confidence. circRNAs found to undergo m6A modification with very high confidence or high confidence were selected for further confirmation.

## Results

### Overview of circrna profiles

**Fig. 1:**
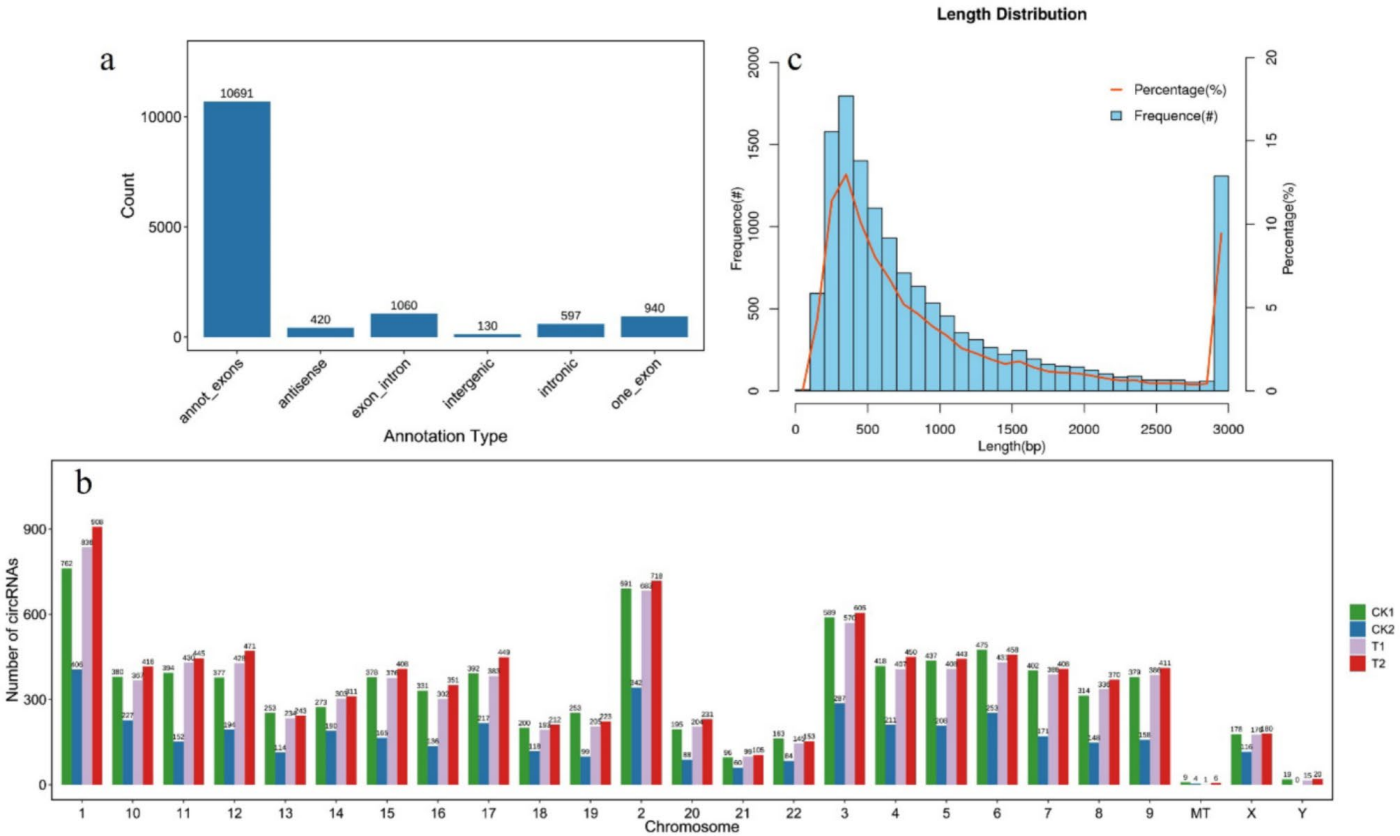
Overview of DECs identified in the CK1, CK2, T1 and T2 groups. (**a**) The bar graph shows the categories of all identified circRNAs. Most circRNAs originated from annotated exons. A small proportion of circRNAs were derived from exons or introns, whereas very few were derived from intergenic regions. (**b**) The bar graph shows the distribution of all identified circRNAs on human chromosomes. (**c**) circRNAs that were 100–800 nt in length accounted for more than half of all circRNAs. circRNA, circular RNA; DECs, differentially expressed circRNAs.

To find potential biomarkers or treatment targets and specifically mention the goals of understanding differences between Ph^+^ and Ph^−^ disease and changes with treatment, high-throughput sequencing of bone marrow aspirates from ALL patients was performed, and then genome-wide maps of circRNAs expressed in children with Ph^−^ B-ALL at diagnosis and in children with Ph^+^ ALL group at diagnosis and two time points after treatment were constructed. While almost all the circRNAs originated from annotated exons, some originated from exons or introns, and a few originated from other regions (Fig. 1a). In addition, circRNAs were found on all chromosomes; the distribution of circRNAs among the different chromosomes varied, but chromosomes 1, 2 and 3 contained the largest number of DECs (Fig. 1b). The number of circRNAs in the Ph^−^ group (CK2) is approximately half that in the Ph^+^ groups across all chromosomes. Next, we assessed the length of the circRNAs and found that more than half of them were 100–800 nt long (Fig. 1c).

### Differences in circrna expression profiles in bone marrow and bioinformatic analysis of DECs between children with ph+^+^ and ph+^−^ B-ALL at diagnosis

#### Identification of DECs

**Fig. 2:**
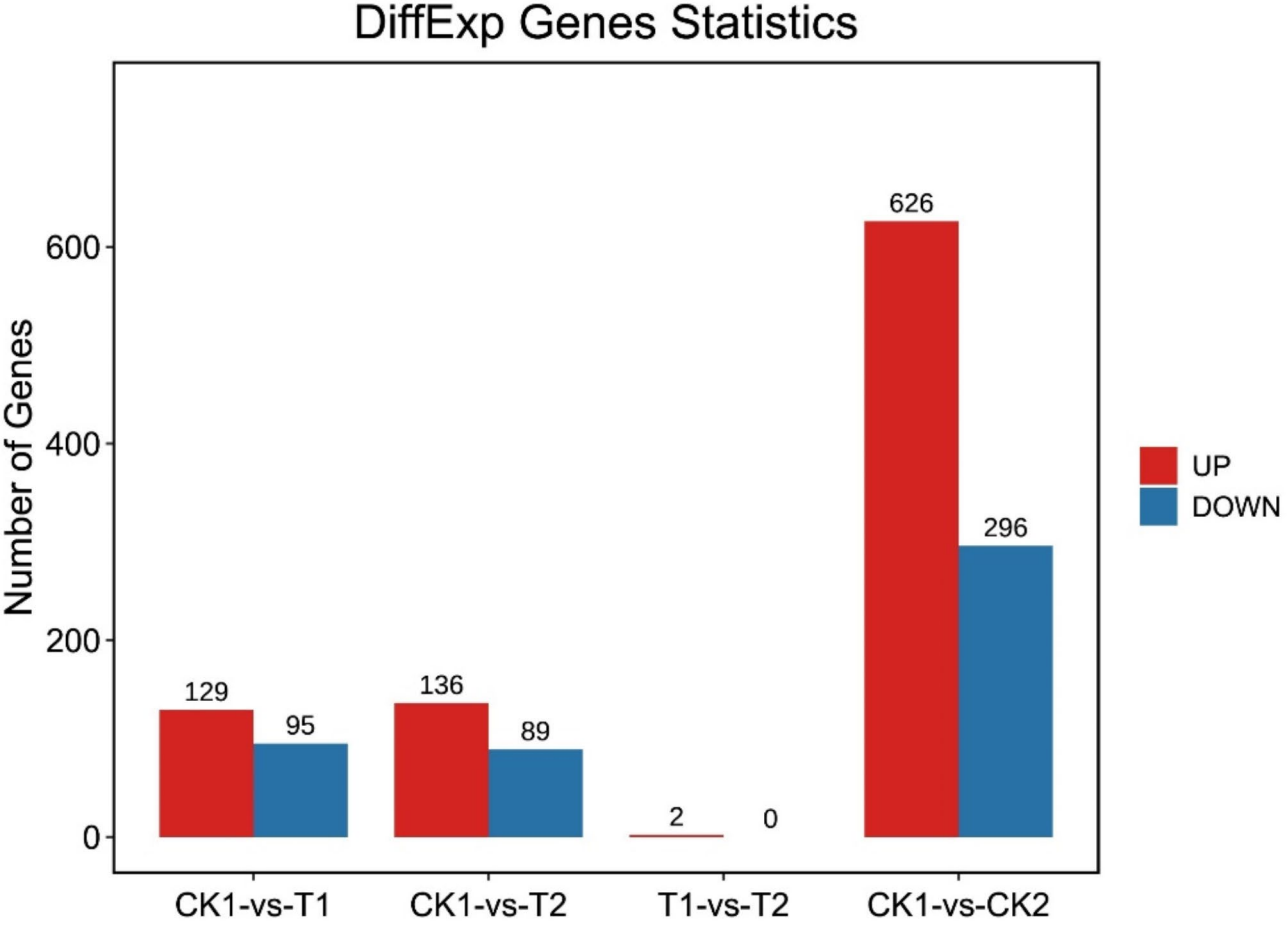
CircRNA expression profiles of the Ph^+^ ALL groups (CK1, T1 and T2 groups) and the control group (CK2 group). circRNA, circular RNA.

Using a corrected P value < 0.05 as the cut-off, we identified DECs in the bone marrow of Ph^+^ B-ALL patients at diagnosis (CK1 group) and a Ph^−^ B-ALL patient at diagnosis (CK2 group). We identified a total of 922 DECs, including 626 upregulated DECs and 296 downregulated DECs, between the CK1 and CK2 groups (Fig. 2). The top 10 upregulated and downregulated DECs are shown in Table 3.

**Table 3:** Top 10 DECs in bone marrow samples collected from ph+^+^ B-ALL patients at diagnosis (CK1 group) compared bone marrow samples collected from ph+^−^B-ALL patients at diagnosis (CK2 group).

Circ-Base ID	*p* value	False-discovery rate	Fold change	CircRNA type
hsa_circ_000525	1.18E−10	4.95E−08	19.68958	exon_intron
hsa_circ_0017627	7.65E−06	0.001027	18.68957589	one_exon
hsa_circ_0032116	7.65E−06	0.001027	18.68958	annot_exons
hsa_circ_0084941	3.06E−05	0.003183	18.49693	annot_exons
hsa_circ_0061290	3.06E−05	0.003183	18.49693	intronic
hsa_circ_0091585	3.06E−05	0.003183	18.49693	annot_exons
hsa_circ_001323	3.06E−05	0.003183	18.49693	annot_exons
hsa_circ_0008945	3.06E−05	0.003183	18.49693	antisense
hsa_circ_0055236	0.000122	0.007921	18.27454	one_exon
hsa_circ_0025630	0.000122	0.007921	18.27454	annot_exons
hsa_circ_0061406	0.000122	0.007921	18.27454	annot_exons
hsa_circ_0012152	1.16E−17	9.73E−15	−21.0385	one_exon
hsa_circ_0009024	5.25E−11	2.43E−08	−20.4888	one_exon
hsa_circ_000913	2.51E−09	7.75E−07	−20.2738	annot_exons
hsa_circ_0006602	2.51E−09	7.75E−07	−20.2222	one_exon
hsa_circ_000455	4.35E−09	1.22E−06	−20.2106	intronic
hsa_circ_0006382	3.48E−07	7.68E−05	−19.864	annot_exons
hsa_circ_0004853	6.44E−07	0.000124	−19.6031	annot_exons
hsa_circ_000722	2.71E−06	0.00044	−19.4485	antisense
hsa_circ_0078299	8.55E−06	0.001131	−19.2614	one_exon
hsa_circ_001644	1.14E−05	0.001466	−19.2598	annot_exons

#### Functional enrichment and pathway analyses of DECs in the bone marrow of ALL patients

**Fig. 3:**
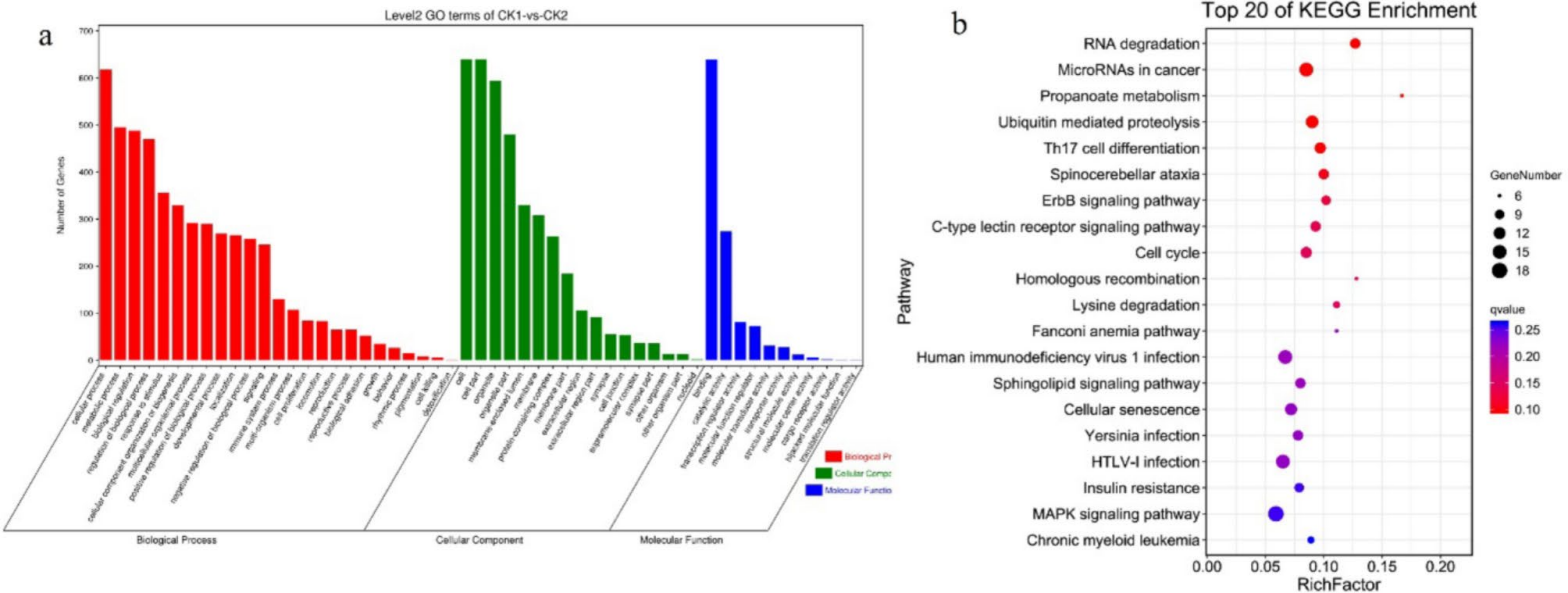
GO enrichment and KEGG pathway analysis of the genes associated with the DECs. GO enrichment analysis involved enrichment analyses of BP, CC, and MF terms. *BP* biological process, *CC* cellular component, *MF* molecular function. (**a**) The most significantly enriched GO terms for DECs between samples collected from children with Ph^+^ ALL at diagnosis (CK1 group) and a sample collected from a child with Ph^−^ B-ALL at diagnosis (CK2 group). (**b**) The most significantly enriched pathways for DECs between samples collected from children with Ph^+^ ALL at diagnosis (CK1 group) and a sample collected from a child with Ph^−^ B-ALL at diagnosis (CK2 group). *circRNA* circular RNA, *GO* Gene ontology, *KEGG* Kyoto Encyclopedia of Genes and Genomes, *Ph* Philadelphia chromosome, *B-ALL* B-cell acute lymphoblastic leukaemia.

Gene Ontology (GO) enrichment and Kyoto Encyclopedia of Genes and Genomes (KEGG)^10–12^ pathway analyses were used to identify the biological and pathophysiological functions of the DECs in children with Ph^+^ B-ALL. GO enrichment analysis of the DECs revealed that the most highly enriched terms included the cellular component (CC) terms intracellular part and organelle; the molecular function (MF) terms regulation of binding and catalytic activity and transcription regulator activity; and the biological process (BP) terms cellular process, metabolic process, biological regulation and regulation of biological process (Fig. 3a). The most highly enriched pathways for the DECs between the CK1 and CK2 groups were RNA degradation, microRNAs in cancer, propanoate metabolism and ubiquitin-mediated proteolysis (Fig. 3b). Many of these pathways are involved in regulating the development of Ph^+^ B-ALL.

### Differences in circrna expression profiles and bioinformatic analysis of DECs among children with ph+^+^ ALL at diagnosis and at two time points after treatment

#### CircRNA expression profiles in bone marrow

We assessed circRNA expression in the bone marrow of Ph^+^ ALL patients at diagnosis and after treatment. We identified 224 DECs between the CK1 and T1 groups, of which 129 were upregulated and 95 were downregulated in T1. Similarly, a total of 225 DECs were identified, including 136 upregulated circRNAs and 89 downregulated circRNAs, from CK1 to T2 groups. Only 2 DECs were upregulated from T1 to T2 (Fig. 2).

#### Functional enrichment and pathway analyses

**Fig. 4:**
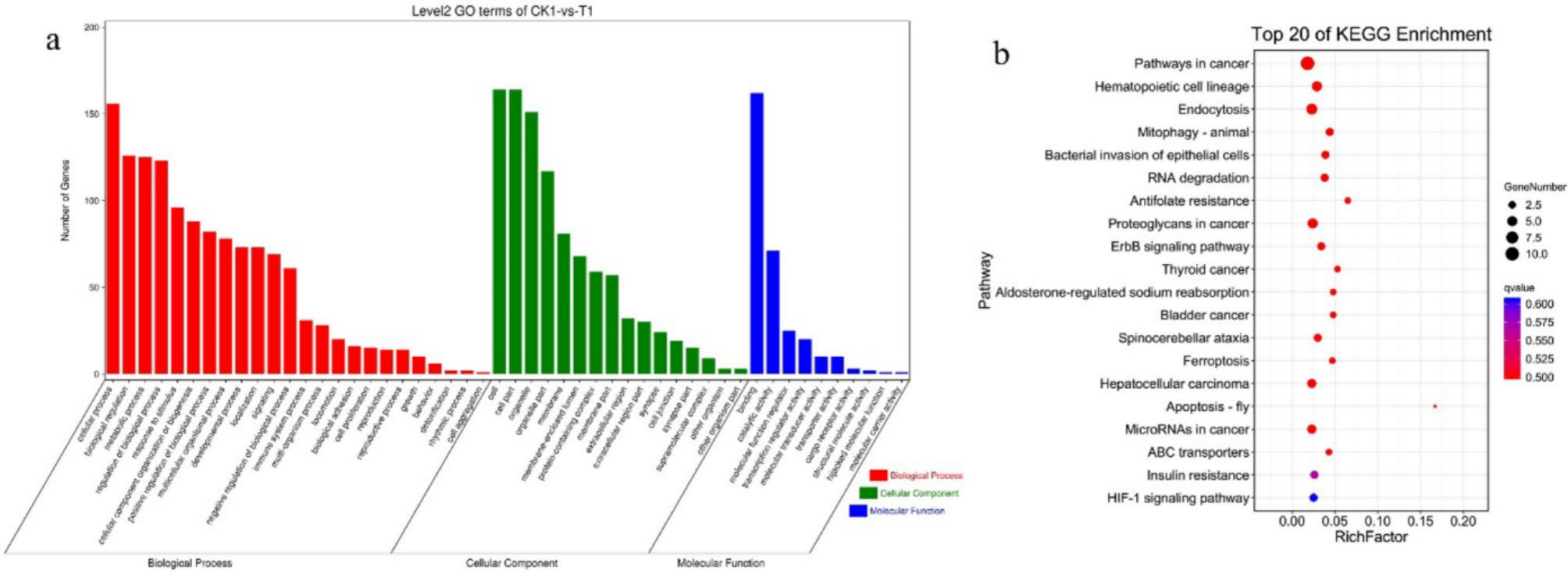
(**a**) The most significantly enriched GO terms for DECs between samples collected from children with Ph^+^ ALL at diagnosis (CK1 group) and samples collected from children with Ph^+^ ALL on day 19 after treatment (T1 group). (**b**) The most significantly enriched pathways for DECs among samples collected from children with Ph^+^ ALL at diagnosis (CK1 group), samples collected from children with Ph^+^ ALL on day 19 after treatment (T1 group). *circRNA* circular RNA, *GO* Gene ontology, *Ph* Philadelphia chromosome, *B-ALL* B-cell acute lymphoblastic leukaemia.

**Fig. 5:**
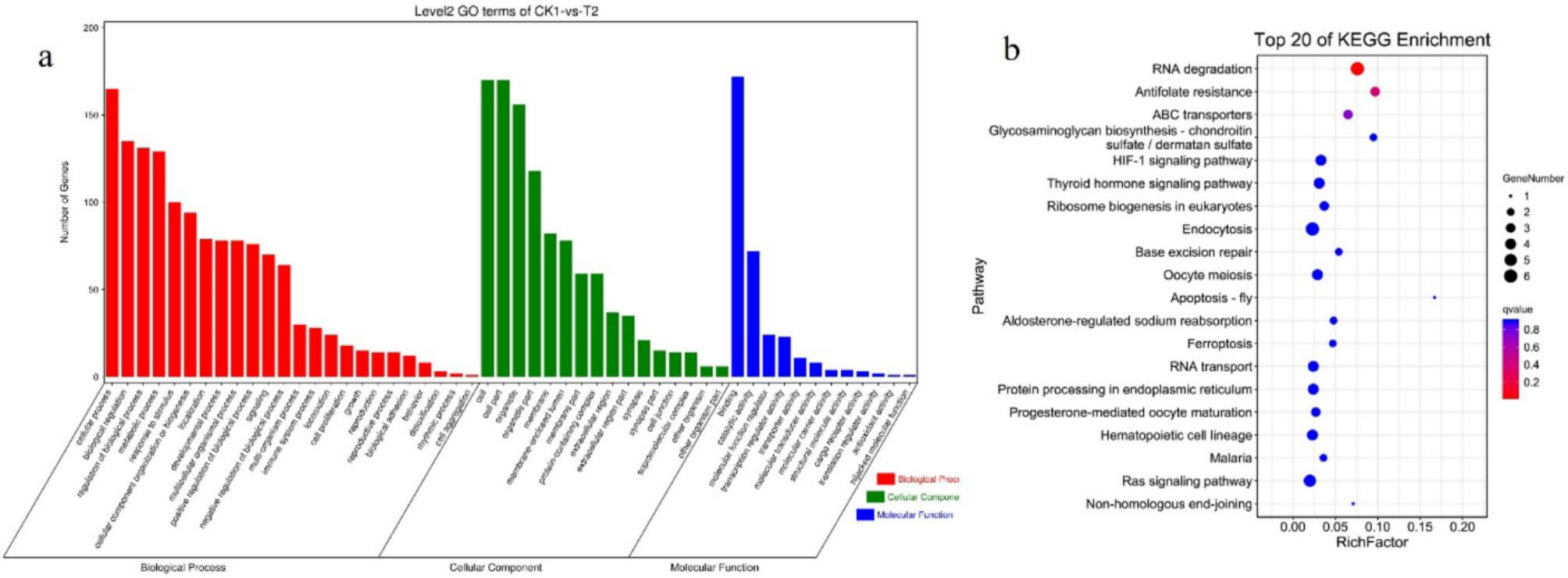
(**a**) The most significantly enriched GO terms for DECs between samples collected from children with Ph^+^ ALL at diagnosis (CK1 group) and samples collected from children with Ph^+^ ALL at first complete remission (day 46 after treatment, T2 group). (**b**) The most significantly enriched pathways for DECs between samples collected from children with Ph^+^ ALL at diagnosis (CK1 group) and samples collected from children with Ph^+^ ALL on day 46 after treatment (T2 group). *circRNA* circular RNA, *GO* Gene Ontology, *CR* complete remission, *Ph* Philadelphia chromosome, *B-ALL* B-cell acute lymphoblastic leukaemia.

GO enrichment and KEGG pathway analyses were used to investigate the potential functions of selected DECs between the CK1 and T1 groups and between the CK1 and T2 groups. The most significantly enriched GO terms in for the DECs between CK1 and T1 were the CC terms cell, cell part, organelle and organelle part; the MF terms binding, catalytic activity and molecular function regulator; and the BP terms cellular process, biological regulation, metabolic process and regulation of biological process (Fig. 4a). Many of these pathways are potential targets for the treatment of Ph^+^ B-ALL. Moreover, GO enrichment analysis revealed that, similar to the DECs between CK1 and T1, the DECs between CK1 and T2 play regulatory roles in cells through various cellular components (Fig. 5a). All of the significantly enriched GO terms for the DECs between CK1 and T2 were related to cell division.

The most highly enriched KEGG pathways for the DECs between the CK1 and T1 groups were pathway in cancer, haematopoietic cell lineage, endocytosis and mitophagy (Fig. 4b), whereas the most highly enriched KEGG pathways for the DECs between the CK1 and T2 groups were RNA degradation and antifolate resistance (Fig. 5b).

### Venn diagram of DECs

**Fig. 6:**
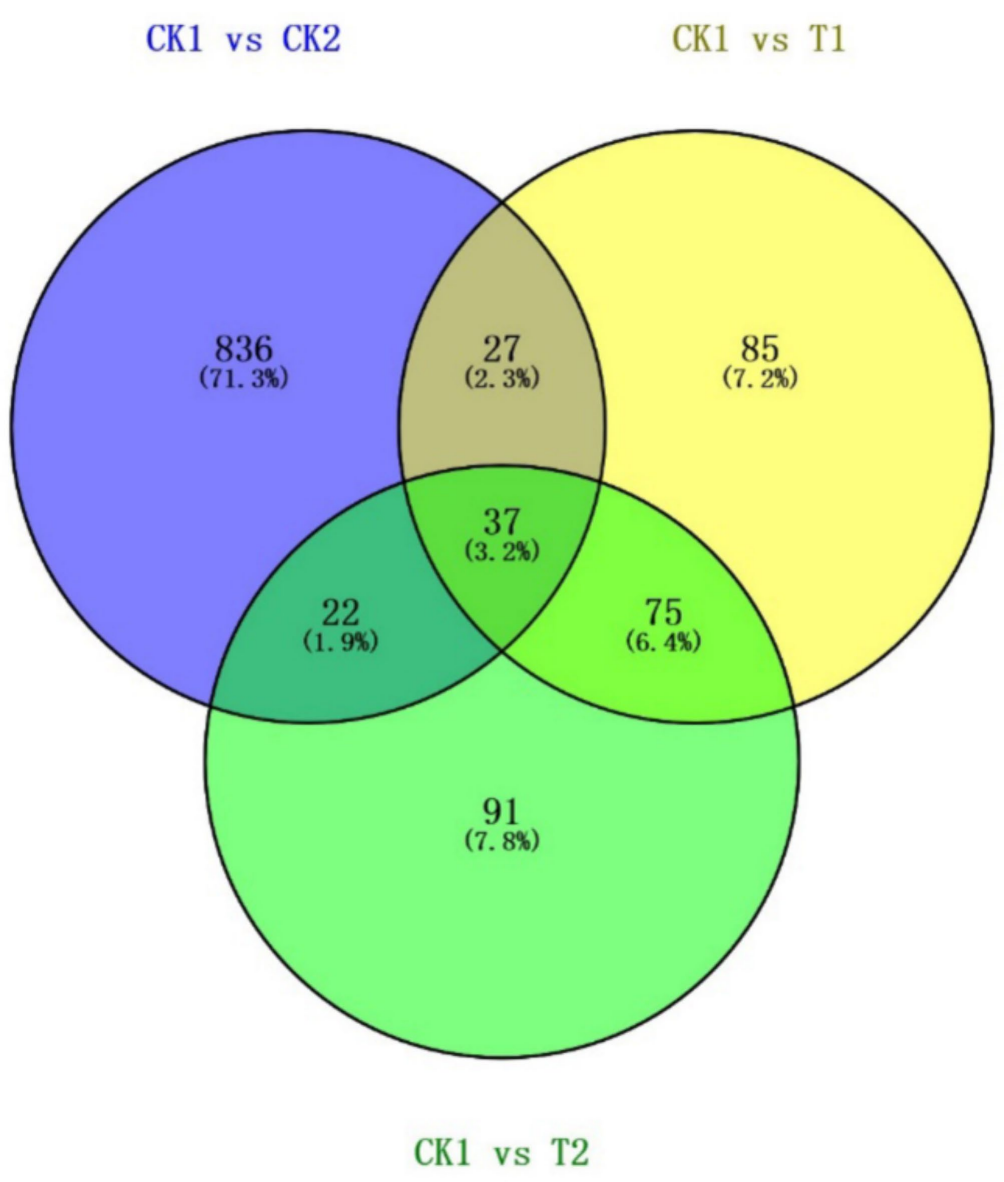
Venn diagrams of the DECs among the three comparisons were constructed. A total of 37 overlapping circRNAs were identified among the comparisons between the CK1 and T1 groups, the CK1 and T2 groups and the CK1 and CK2 groups. DECs, differentially expressed circular RNAs; circRNA, circular RNA.

Venn diagrams of the DECs from the three comparisons (the CK1 group vs. the T1 group, the CK1 group vs. the T2 group, and the CK1 group vs. the CK2 group) were constructed (Fig. 6) via Venny 2.1 (https://bioinfogp.cnb.csic.es/tools/venny/) to identify intersecting circRNAs. A total of 37 overlapping circRNAs were identified among the comparisons between the CK1 and T1 groups, the CK1 and T2 groups and the CK1 and CK2 groups (Fig. 6). These results support our other data, suggesting that circRNAs may play a role in the onset of childhood ALL and the treatment of Ph^+^ ALL in children, but further investigation is warranted.

### Verification of changes in circrna expression

**Fig. 7:**
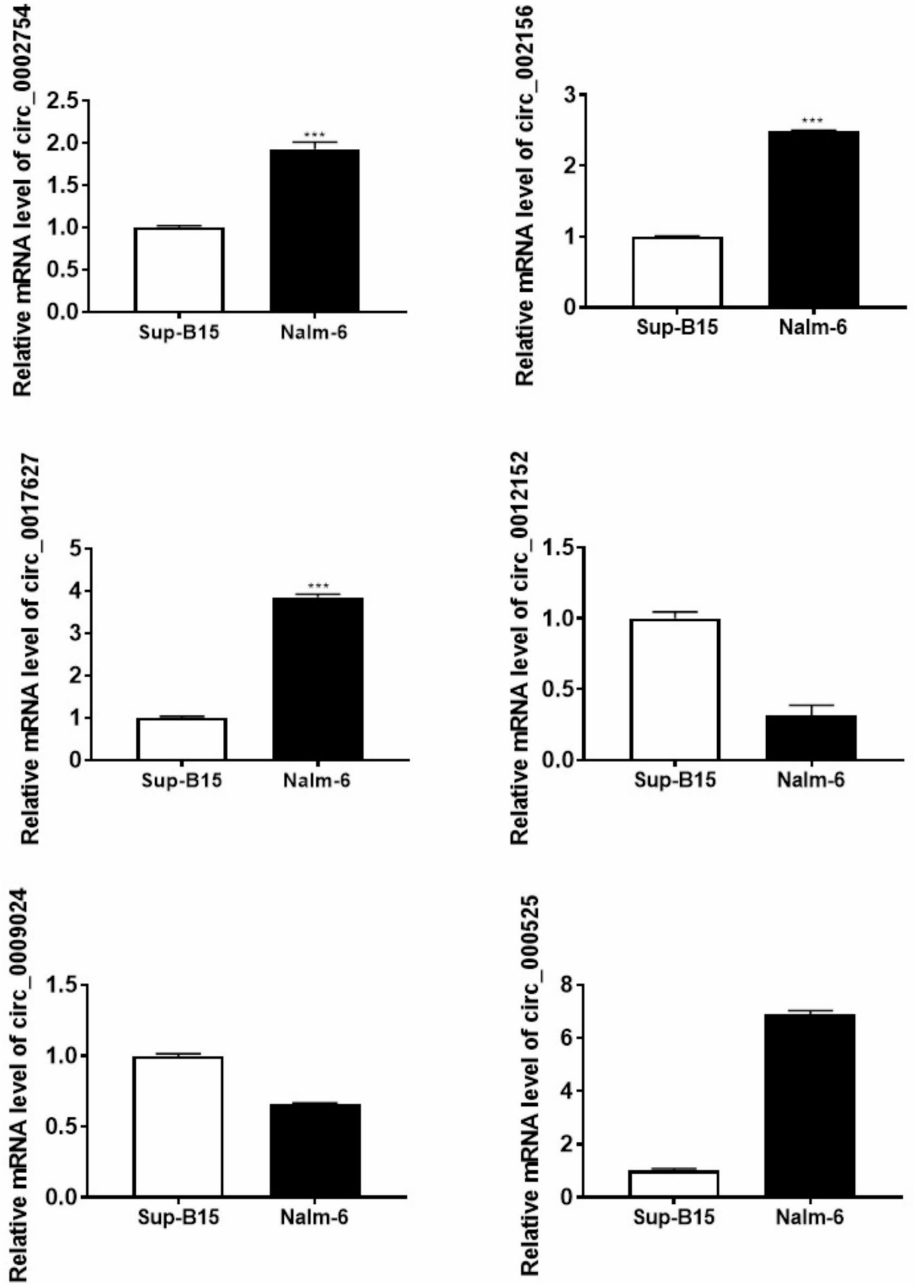
Validation of DEC expression in B-ALL cell lines via qRT‒PCR. The expression levels of five selected circRNAs in a Ph^+^ B-ALL cell line (Sup-B15) and a Ph^−^ B-ALL cell line (Nalm-6). *circRNA* circular RNA, *B-ALL* B-cell acute lymphoblastic leukaemia, *PCR* polymerase chain reaction, *Ph* Philadelphia chromosome.

To verify our DEC data, the expression levels of three upregulated circRNAs (hsa_circ_0012152, hsa_circ_0009024 and hsa_circ_0002754) and three downregulated circRNAs (hsa_circ_002156, hsa_circ_0017627 and hsa_circ_000525) were verified in a Ph^+^ B-ALL cell line (Sup-B15 cell line) and a Ph^−^ B-ALL cell line (Nalm-6 cell line) by qRT‒PCR. The expression levels of hsa_circ_002156, hsa_circ_0002754 and hsa_circ_0017627 in the Ph^−^ B-ALL cell line were significantly greater than those in the Ph^+^ B-ALL cell line (*P* < 0.001). The expression levels of hsa_circ_0012152 and hsa_circ_0009024 in the Ph^+^ B-ALL cell line were greater than those in the Ph^−^ B-ALL cell line. The expression level of hsa_circ_000525 in the Ph^−^ALL cell line was greater than that in the Ph^+^ B-ALL cell line. Thus, the expression patterns of the six selected DECs in B-ALL cell lines were generally consistent with those in the bone marrow of children with B-ALL revealed by the high-throughput sequencing data, demonstrating the reliability of our circRNA sequencing data (Fig. 7). However, the expression level of hsa_circ_0002754 in the Ph^−^ B-ALL cell line was greater than that in the Ph^+^ B-ALL cell line, which was not concordant with the sequencing data. The inconsistency between the PCR results and the sequencing results (including the data mining results) regarding hsa_circ_0002754 expression may be related to genetic factors or issues with the sequencing process. In conclusion, samples collected from Ph^+^ ALL patients at diagnosis presented circRNA expression patterns that were distinct from those observed in samples collected from Ph^−^ ALL patients at diagnosis and from Ph^+^ ALL patients after treatment according to both transcriptome data and qRT‒PCR verification.

### Analysis of m6A modification of the DECs

**Fig. 8:**
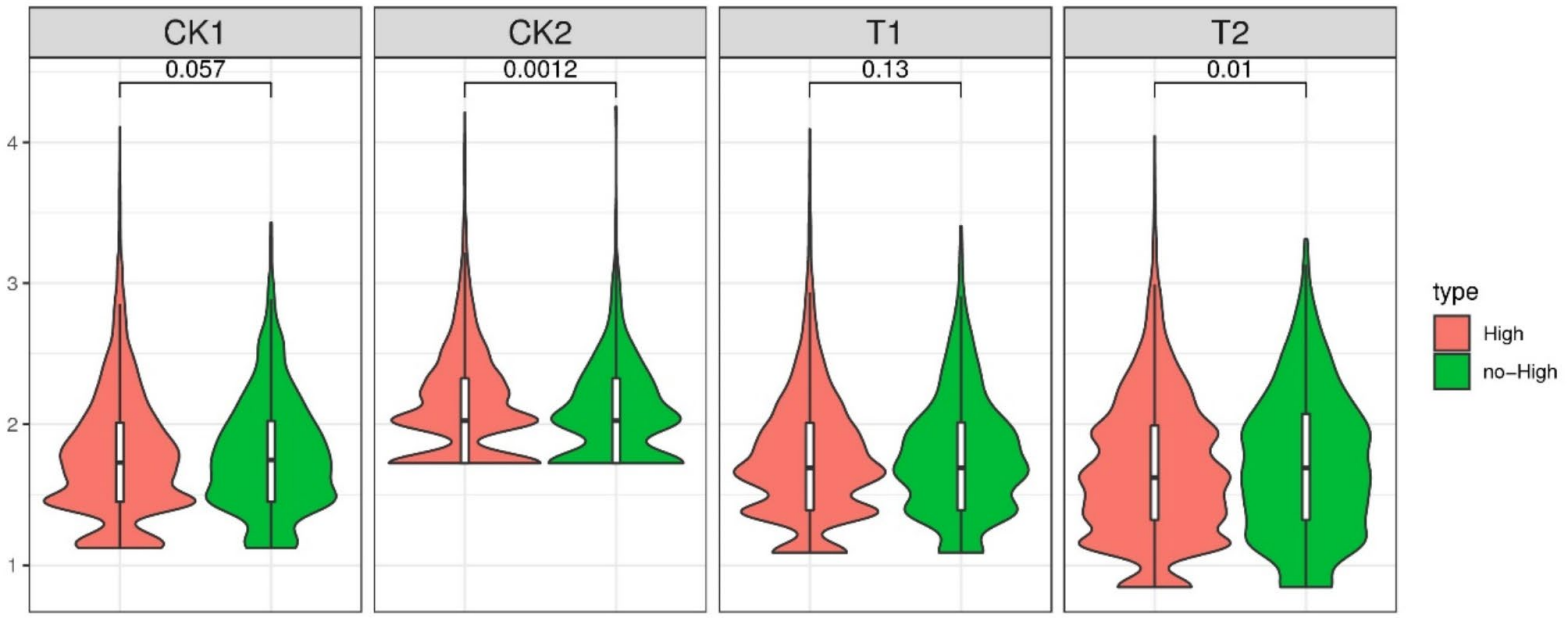
The expression levels of circRNAs with high- and very high-confidence m6A sites were compared among the different groups (CK1, CK2, T1, and T2 groups), and a violin diagram was drawn, with the horizontal axis representing the group, and the vertical axis representing the circRNA expression level. circRNAs with an expression level of 0 were removed, and the log10 values of expression levels are shown. *circRNA* circular RNA, *m6A* N6-methyladenosine.

m6A modification is the most prevalent posttranscriptional RNA modification, that accelerates RNA degradation and thereby fine-tunes gene expression and is involved in fine-tuning gene expression^13^. Therefore, to explore the role of the m6A modification of circRNAs in children with Ph^+^ ALL, the potential m6A sites of DECs were predicted via SRAMP (http://www.cuilab.cn/sramp/)^14^. The thresholds for very high-, high-, moderate-, and low-confidence m6A sites were specificities of 99%, 95%, 90%, and 85% (i.e., false-positive rates of 1%, 5%, 10%, and 15%) on cross-validation tests, respectively. According to the level of confidence in the data, the circRNAs were divided into two categories: those with high or very high confidence m6A modification sites (additional file: high.circRNA.result) and those without such sites (additional file: nohigh.circRNA.result). The expression levels of circRNAs from these two categories were compared among the CK1, CK2, T1, and T2 groups, and a violin diagram was drawn, with the horizontal axis representing the group and the vertical axis representing the circRNA expression level (circRNAs with an expression level of 0 were removed, and the log10 values of the expression level data are shown) (Fig. 8).

## Discussion

CircRNAs are exceptionally stable noncoding RNAs. Some circRNAs have been described as efficient miRNA sponges with the potential to regulate gene expression, including the expression of genes involved in cancer^15^. However, few studies have focused on the role of circRNAs in ALL, and the function and regulatory mechanism of circRNAs in Ph^+^ B-ALL are unknown. In this study, we identified circRNAs with large differences in expression between Ph^+^ B-ALL patients and Ph^−^ B-ALL patients at diagnosis and among Ph^+^ B-ALL patients at diagnosis and at two time points after treatment. To our knowledge, this is the first study involving global expression analysis and bioinformatic analyses of circRNAs in childhood Ph^+^ B-ALL.

Chromosomal translocations associated with cancer give rise to fusion circRNAs, which have been shown to contribute to cellular transformation, affect cell viability, and promote tumour development in in vivo models^16^. Our results indicate the existence of a unique circRNA expression signature associated with Ph^+^ B-ALL pathogenesis. Analysis of highly significant DECs revealed large differences among Ph^+^ and Ph^−^ B-ALL patients before treatment and among Ph^+^ B-ALL patients before and after treatment. The differences in expression of the DECs between the CK1 and T1 groups and between the CK1 and T2 groups were less significant than the differences in expression of the DECs between the CK1 and CK2 groups. More interestingly, only 2 DECs were detected between the T1 and T2 groups. Further, our results indicate that there are more m6A-modified circRNAs in Ph^+^ ALL than in Ph^−^ ALL. The trends of the variations in circRNA expression were consistent with the clinical features of the patients. The fact that we found many DECs among the groups indicate that circRNAs are likely to contribute to the occurrence and development of Ph^+^ B-ALL, extending our knowledge regarding circRNA expression in Ph^+^ B-ALL.

Moreover, we also found that the DECs in the bone marrow of B-ALL patients were encoded mainly by exonic regions in the human genome, indicating that exonic regions may act as major sources of circRNAs in human bone marrow. The DECs were widely distributed among various human chromosomes, which is consistent with previous reports.

To confirm the deep sequencing data, the expression of six randomly selected DECs in cell lines was verified via qRT‒PCR. We found that the expression levels of all the selected circRNAs in the cell lines was consistent with the expression data from B-ALL patient tissues. However, the expression level of hsa_circ_0002754 in the ALL-cell lines did not closely match the expression level in B-ALL patient tissues according to the sequencing results, which might have been due to genetic variations in the patients from which the cell lines were derived.

Analysis of the distribution of the DECs on human chromosomes revealed that chromosomes 1, 2 and 3 contained the most DECs in B-ALL patients, which suggested that chromosomes 1, 2 and 3 may be involved in tumorigenesis in B-ALL. Several previous studies have confirmed the critical role of chromosomes 1, 2 and 3 in haematological malignancies. As chromosome 1 is the largest human chromosome and contains over 1600 known genes and 1000 novel coding sequences or transcripts, it is not surprising that recurrent chromosome 1 abnormalities are common in both neoplastic and nonneoplastic diseases^17^. Another study revealed that a hemizygous deletion on mouse chromosome 2 (del2) is a common feature in several mouse strains susceptible to radiation-induced AML (rAML)^18^. Structural and numerical aberrations of chromosome 3 have been identified in haematological diseases. Aberrations of chromosome 3 are found in certain regions, 3p14-21, 3q21 and 3q26, in leukaemias, suggesting that these aberrations are nonrandom events important for the development of the malignancy^19^. ALL patients with abnormalities in chromosome 3 may have unfavourable outcomes^20^. These observations suggest that certain chromosome 1, 2 and 3 regions might harbour oncogenes or tumour suppressor genes that are pathogenetically relevant to B-ALL. Therefore, circRNAs derived from chromosomes 1, 2 and 3 may play a very important role in diseases by regulating the expression of target genes.

GO enrichment and KEGG pathway analyses to determine the potential functions of the DECs revealed that the GO terms in which the DECs were most highly enriched were closely associated with leukaemia and haematologic and immune system cancers. The most highly enriched pathways for the DECs were directly related to human T lymphotropic virus 1 (HTLV-1) infection, cellular senescence and the cell cycle. Moreover, Reactome pathway analysis revealed that the DECs were closely associated with the cell cycle, mitosis, gene expression (transcription) and posttranslational protein modification. Thus, the results indicated that the identified DECs play a key role in the cell cycle. In cancer cells, various cell cycle pathways are aberrantly regulated, and dysregulation of cell cycle-related gene expression can cause uncontrolled cancer cell proliferation. CircRNAs act as tumour suppressors or oncogenes in the development of AML and are emerging as new diagnostic and prognostic biomarkers^21^, which is consistent with our results. More investigations should be performed to characterize the exact function of circRNAs in childhood ALL, especially Ph^+^ ALL, to identify biomarkers with diagnostic, prognostic or therapeutic potential for ALL. Together, our results indicate that the identified DECs might play a role in regulating childhood B-ALL progression.

M6A modification is one of the most prevalent RNA modifications in higher eukaryotes. Emerging evidence suggests that m6A modification plays important and diverse biological roles in tumorigenesis, invasion, metastasis and other processes^22^. However, to date, there have been no conclusive studies on circRNA expression in childhood B-ALL. In our study, we measured the m6A modification of DECs in the bone marrow of B-ALL patients. We found that m6A modification was increased in Ph^+^ ALL patients compared with Ph^−^ ALL patients. Previous studies have revealed that m6A modification can drive the extensive translation of circRNAs, suggesting that the m6A modification of circRNAs may control circRNA functionality^23,24^. Our results reveal the potential m6A sites of the identified DECs. We identified several high- and very high-confidence m6A sites in DECs in the CK1 group. There was a significant difference in the number of high- and very high-confidence m6A sites in DECs between the CK1 and CK2 groups. Overall, our results suggest that the m6A modification of DECs may be prevalent in children with Ph^+^ B-ALL and may be closely related to circRNA functionality, thereby providing new insights into the regulation of circRNA function through m6A modification in children with Ph^+^ B-ALL.

There are several limitations in our study. First, the sample size was relatively small, and the difference in the number of samples between the CK1 and CK2 groups limits the applicability of the study findings; therefore, our results may not be fully generalizable. In addition, while we confirmed the expression levels of selected DECs using qRT‒PCR, further validation is necessary to establish the reliability of these circRNAs as diagnostic biomarkers. Additional functional studies will also be important to elucidate the mechanisms underlying the roles of these circRNAs in B-ALL and the functions of m6A-modifications of the DECs. We are now attempting to study the effects of the m6A modification of the identified DECs in childhood B-ALL and the mechanisms underlying these effects. We will explore other types of epigenetic modifications or extend the study to other types of leukaemia and collect more clinical samples to study the relationships among these circRNAs. Conducting longitudinal studies that track circRNA expression changes over the course of treatment and disease progression will provide invaluable information regarding the role of circRNAs as potential biomarkers for monitoring disease status and the therapeutic response.

## Conclusion

We assessed the global expression of circRNAs in the bone marrow of children with B-ALL and identified DECs between Ph^+^ B-ALL patients and Ph^−^ B-ALL patients at diagnosis and between Ph^+^ B-ALL patients at diagnosis and Ph^+^ B-ALL patients after treatment. The results indicate that circRNAs may be involved in the pathogenesis of Ph^+^ B-ALL. Moreover, we identified m6A-modified DECs in Ph^+^ B-ALL samples, providing new insights into the involvement of circRNAs and m6A modification in Ph^+^ B-ALL. Our observations expand the current knowledge about the onset and progression of ALL and reveal new avenues for potential therapeutic interventions.

## Data Availability

The datasets generated and analysed during the current study are available in the Genome Sequence Archive repository, at the National Genomics Data Center, Beijing Institute of Genomics, Chinese Academy of Sciences/China National Center for Bioinformation, https://ngdc.cncb.ac.cn/bioproject/browse/PRJCA015601.
